# Dataset on Cs-137 in waters surrounding Taiwan

**DOI:** 10.1016/j.dib.2023.109758

**Published:** 2023-11-07

**Authors:** Ting-Hsuan Huang, Ming-Ta Lee, Wei-Jen Huang, Liang-Yu Tao, Ming-An Lee, Sen Jan, Yiing-Jang Yang, Chen-Tung Arthur Chen

**Affiliations:** a Department of Oceanography, National Sun Yat-sen University, Kaohsiung 804, Taiwan; b Taiwan Ocean Research Institute, National Applied Research Laboratories, Kaohsiung 852, Taiwan; c Radiation Monitoring Center, Atomic Energy Council, Kaohsiung 833, Taiwan; d Department of Environmental Biology and Fisheries Science, National Taiwan Ocean University, Keelung 202, Taiwan; e Institute of Oceanography, National Taiwan University, Taipei 106, Taiwan

**Keywords:** Fukushima accident, Kuroshio, Taiwan strait, South China sea, East China Sea

## Abstract

The Fukushima accident released short-lived Cs-134 and longer-lived Cs-137 to the ocean. The amount, although substantial, is much less than that produced during the atomic bomb tests 60 yrs ago. Cs-134 and Cs-137 are anthropogenic radionuclides and soluble in seawater; hence, the radioactivity can be used as a tracer for special events or currents. Samples of Cs-134 and Cs-137 in seawater were collected around Taiwan, including the Kuroshio, the northern South China Sea, the Taiwan Strait, and the southern East China Sea from 2018 to 2021. The average surface Cs-137 activity was 1.18±0.25 Bq *m* ^−^ ^3^, and the activities of Cs-134 samples were all under the detection limit. Complete data are archived, including sampling date, location, water depth, temperature, salinity, and Cs-137 activity; the total sample amount is 577.

Specifications Table

**Table:** 

Subject	Environmental Science/Hydrology and Water Quality
Specific subject area	Spatial and vertical distribution of Cs-137 in seawaters surrounding Taiwan, including the Kuroshio, the northern South China Sea, the Taiwan Strait, and the southern East China Sea from 2018 to 2021.
Data format	Raw
Type of data	Table, Figure
Data collection	Seawater samples were taken from ships of opportunity. Surface samples were taken from a bucket, and subsurface samples were taken from a CTD Rosette.
Data source location	· City/Town/Region: Kaohsiung · Country: Taiwan · Latitude and longitude for collected samples/data: various latitudes and longitudes as listed in the table · Institution: Radiation Monitoring Center, Atomic Energy Council
Data accessibility	Repository name: PANGAEA Data identification number: 10.1594/PANGAEA.949281 Direct URL to data: https://doi.pangaea.de/10.1594/PANGAEA.949281
Related research article	C.T.A. Chen, T.H. Huang, W.J. Huang, Y.J. Yang, S. Jan, M.A. Lee, and M.T. Lee, The Kuroshio radiocesium stream (2022), Mar. Pollut. Bull. 182, 114,026 [1].

## 1 Value of the Data

• The most comprehensive Cs-137 data for waters near the origin of the Kuroshio are provided.
• Academics and governments can use this dataset for constructing research plans and long-term monitoring.
• The temporal and spatial distributions of Cs-137 contribute to the ongoing study of the Fukushima incident impacts and ocean current research.

## 2 Data Description

**Fig. 1:**
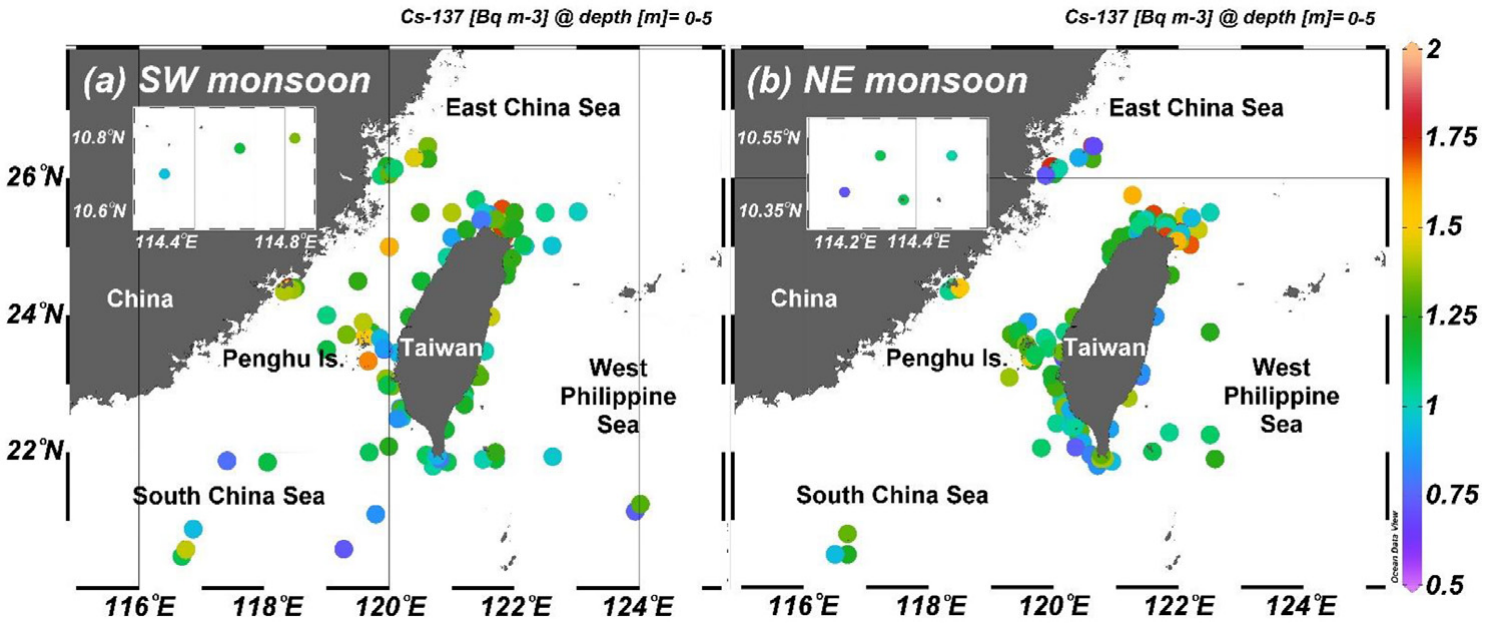
Sampling locations and surface water Cs-137 activities in the a) SW and b) NE monsoon seasons.

**Fig. 2:**
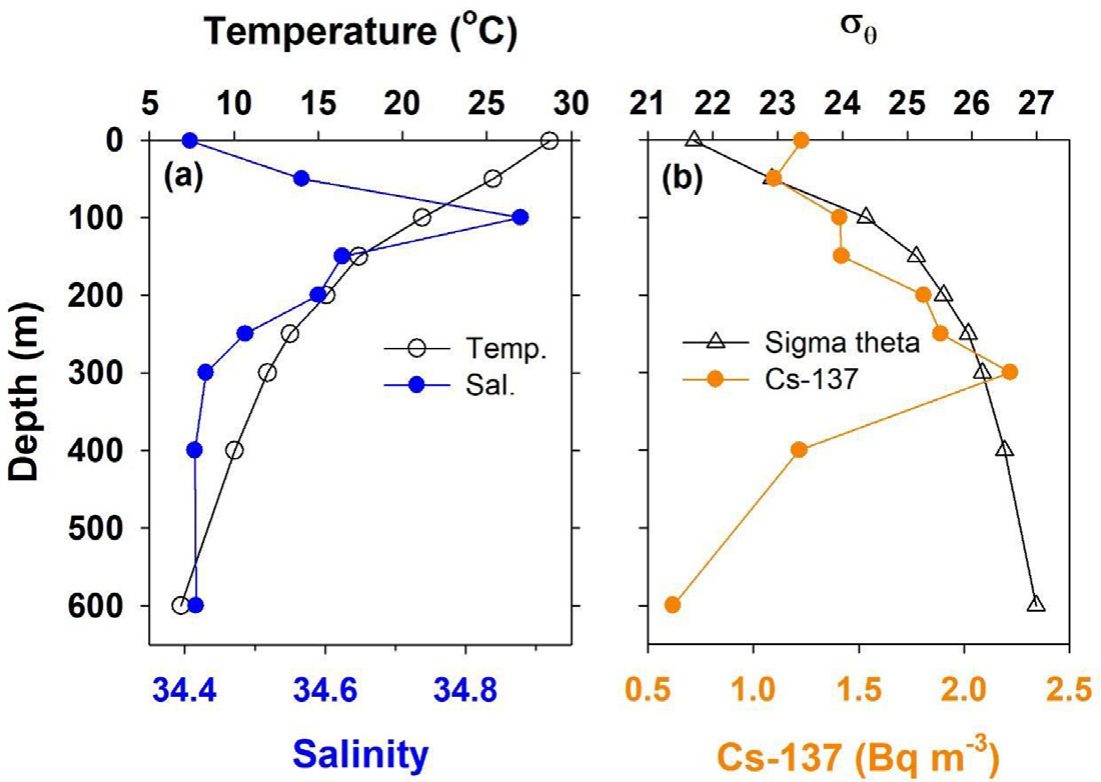
The vertical distributions of a) temperature and salinity vs. depth, and b) Cs-137 activity and σ_θ_ vs. depth.

The dataset of Cesium-137 (Cs-137) activity in waters surrounding Taiwan also includes sampling date, location, water depth, temperature, and salinity, and the table title and parameter units are listed in Table 1. A total of 577 samples were collected from 2018 to 2021, and 170 out of 577 were sampled deeper than 5 m. The sampling locations were between 10 and 27˚N and 114–124˚E[2]. Fig. 1 shows the surface Cs-137 distributions during the SW monsoon (May to September) and NE monsoon seasons (October to April), and the surface sample numbers are 166 and 241, respectively. The average Cs-137 activity was 1.18±0.25 Bq *m* ^−^ ^3^. Fig. 2 shows typical vertical distributions of T, S, sigma t, and Cs-137 activity. The temperature decreases with water depth, while the salinity shows a maximum subsurface value. The sigma theta increases with depth. The Cs activity increases with depth initially to reach a subsurface maximum of 2.4 Bq *m* ^−^ ^3^ at about 300 m.

**Table 1:** Format of table.

Column	Parameter	Unit
1st	Sampled year	–
2nd	Sampled month	–
3rd	Sampled day	–
4th	Latitude	˚N
5th	Longitude	˚E
6th	Water depth	m
7th	Temperature	°C
8th	Salinity	–
9th	Cs-137 activity	(Bq *m* ^−^ ^3^)

## 3 Experimental Design, Materials and Methods

Cs-137 is produced by nuclear fission as one of the anthropogenic radionuclides. Through nuclear weapon testing and nuclear accidents, the nuclear fallout and wastes released easily-dissolved Cs-137 into the oceans [2]. On March 11, 2011, a magnitude nine major earthquake followed by a tsunami destroyed Japan's Fukushima Daiichi Nuclear Power Plant, resulting in the most severe nuclear accident since the Chernobyl disaster in 1986. Within two weeks, the atmospheric circulation quickly transported radioactive material released into the air throughout the northern hemisphere. Short-lived Cs-134 (with a half-life of 2.0648 yrs) and longer-lived Cs-137 (with a half-life of 30.17 yrs) were detected in aerosols over Taiwan on March 25, 2011. The Fukushima accident also released a tremendous amount of radioactive material into adjacent seawater. Since Cs-137 is soluble in seawater, the pattern of varying concentrations can be used as tracers for the current flow and specific point sources. In 2002, Aoyama et al. [3] identified the Cs-137 signal in the western North Pacific as the global fallout of Cs during the atomic bomb tests in the late 1950s and early 1960s. Recently, the distribution of Cs-137 around Taiwan was evaluated, revealing its origin from the atomic bomb tests [1]. This article adds value to the published work by providing specifications and link to the raw data so that interested investigators could make further contributions.

To understand the activity distributions of Cs-134 and Cs-137 in the seawater around Taiwan, surface (< 5 m) and subsurface seawater samples were collected at sites in the Kuroshio east of Taiwan, the northeastern South China Sea, the Taiwan Strait, and the southern East China Sea (Fig. 1) during 2018 to 2021. Surface seawater samples (40 or 60 L) were collected chiefly from fishing boats by using cleaned 20-L tanks. Subsurface samples were taken by using Niskin bottles mounted on a Conductivity–Temperature–Depth (CTD) rosette, which recorded temperature(T), salinity (S; from conductivity), and water depth (from pressure) onboard R/Vs Ocean Researcher I, II, and III. Each 20-L sample was acidified using hydrochloric acid (11 M HCl, 100 mL) and was kept at room temperature (∼15–30 °C) until it was transported to the Radiation Monitoring Center, Atomic Energy Council, Kaohsiung, Taiwan. The sample was initially pre-filtered with a 0.25 mesh filter cotton to remove larger particles, such as sand. Following this, ammonium molybdophosphate (AMP) was utilized to pre-concentrate the radiocesium. The sample was then analyzed using a high-purity germanium (HPGe) detector, which was equipped with lead shielding [4,5]. Each 40-L or 60-L sample was counted for 200,000 s or 120,000 s, respectively. The detection limits of both Cs-134 and Cs-137 are 0.5 Bq *m* ^−^ ^3^. All Cs-134 activities data were not included in the dataset because they were below the detection limit.

## Limitations

Not applicable

## Ethics Statement

The authors declare that there are no ethical issues with the data collection.

## CRediT authorship contribution statement

**Ting-Hsuan Huang:** Writing – original draft, Visualization. **Ming-Ta Lee:** Formal analysis, Resources. **Wei-Jen Huang:** . **Liang-Yu Tao:** Formal analysis, Resources. **Ming-An Lee:** Investigation, Resources. **Sen Jan:** Investigation, Resources. **Yiing-Jang Yang:** Investigation, Resources. **Chen-Tung Arthur Chen:** Writing – review & editing, Supervision.

## Data Availability

Cs-137 in waters surrounding Taiwan from 2018 to 2021 (Original data) (PANGAEA)

## References

[1] Chen C.T.A., Huang T.H., Huang W.J., Yang Y.J., Jan S., Lee M.A., Lee M.T. (2022). The Kuroshio radiocesium stream. Pollution.

[2] Chung Y.C., Chen C.T.A., Nihoul J.C.J., Chen C.T.A. (2007). Encyclopedia of Life Support Systems.

[3] Aoyama M., Hirose K., Nemoto K., Takatsuki Y., Tsumune D. (2008). Water masses labeled with global fallout 137Cs formed by subduction in the North Pacific. Geophys. Res. Lett..

[4] Feldman C., Rains T. (1964). The collection and flame photometric determination of Cesium. Anal. Chem..

[5] Krishnamoorthy T., Doshi G., Sastry V. (1971). Exchange capacity of ammonium phosphomolybdate for caesium by batch technique. Curr. Sci..

